# Intraoperative gastroesophageal reflux in dogs undergoing mastectomy under opioid-based protocols: a preliminary endoscopic study

**DOI:** 10.1007/s11259-026-11290-5

**Published:** 2026-05-27

**Authors:** Priscila dos Santos Ribas, Heytor Jales Gurgel, Gabriela Melo Alves dos Santos, Verena Siqueira da Silva, Francisco Décio de Oliveira Monteiro, Sheyla Farhayldes Souza Domingues, Roberta Martins Crivelaro Thiesen, Roberto Thiesen, Pedro Paulo Maia Teixeira

**Affiliations:** 1 Veterinary Medicine Institute, Federal University of Pará, Castanhal, Pará Brazil; 2 Federal Institute of Tocantins (IFTO), Campus Araguatins, Araguatins, Tocantins Brazil

**Keywords:** Canine mastectomy, Gastroesophageal reflux, Intraoperative endoscopy, Opioid anaesthesia, Lower oesophageal sphincter relaxation, Dog

## Abstract

Gastro‑oesophageal reflux (GER) is a common but underdiagnosed anaesthetic complication in dogs. Although locoregional techniques and other intravenous protocols are available to control pain during mastectomy, the use of opioids and their association with intraoperative GER during this procedure are poorly understood. This preliminary study aimed to evaluate and visualise the possible presence of relaxation of the lower oesophageal sphincter (LES) and gastric reflux in swollen breasts undergoing mastectomy. To achieve this, twelve bitches undergoing unilateral mastectomy underwent oesophageal endoscopy during the procedure to visualise relaxation of the lower oesophageal sphincter (LES) and gastric reflux. The anaesthetic protocol included midazolam combined with morphine or tramadol. Gastro‑oesophageal reflux was observed in 100% (12/12) of bitches during endoscopic monitoring, which began approximately 45 min after induction and continued throughout surgery, with 58% classified as mild and 42% as severe. LES relaxation was observed in all cases. One bitch had pre-existing oesophagitis. The small sample size and the lack of a control group limit generalisability. This preliminary study observed a 100% incidence of intraoperative GER in bitches undergoing mastectomy. However, due to the lack of a non-surgical control group, it was not possible to differentiate whether this was attributable to the surgical procedure itself or the known effects of the opioid-based anaesthetic protocol. These preliminary, hypothesis-generating findings highlight the need for controlled studies to quantify the specific risk associated with mastectomy. Intraoperative vigilance remains essential.

## Introduction

Gastroesophageal reflux (GER) represents a recognised complication of general anaesthesia in dogs, is defined as the passive retrograde movement of gastric contents into the oesophagus. Opioid administration, particularly morphine, reduces lower oesophageal sphincter pressure and increases reflux likelihood (Kraichely et al. 2010; Flouraki et al. 2022a). Patient positioning and breed may also influence intraoperative GER (Wilson et al. 2005; Savvas et al. 2022). In anaesthetized patients, GER is often clinically silent but can lead to significant complications, including oesophagitis, oesophageal stricture formation, and aspiration pneumonia, the latter of which carries a poor prognosis (Galatos and Raptopoulos 1995; Flouraki et al. 2022b).

Mastectomy is frequently performed in female dogs, and while opioids remain commonly used for perioperative analgesia, numerous alternative drugs (e.g., non-steroidal anti-inflammatory drugs, gabapentin, constant rate infusions of lidocaine or ketamine) and locoregional anaesthetic techniques (e.g., tumescent anaesthesia, epidural, or paravertebral blocks) have been described as part of intraoperative and perioperative pain management strategies (Teixeira et al. 2013; Crociolli et al. 2015; Vullo et al. 2021). However, data on intraoperative GER during this procedure are scarce.

The relationship between anaesthetic duration, surgical stimulation, and GER severity has not been systematically examined in the context of mastectomy. Prolonged anaesthesia time may increase gastric content exposure to oesophageal mucosa, while surgical manipulation could potentially influence sphincter relaxation. However, the present study was not designed to evaluate such correlations directly, and any speculation regarding exacerbation of reflux severity by surgical factors would be unwarranted given the absence of a control group. Instead, this observational study simply describes GER occurrence in this specific clinical context.

To address this knowledge gap, we conducted a prospective observational study using intraoperative oesophagoscopy to evaluate the occurrence of GER and lower oesophageal sphincter relaxation in twelve bitches undergoing unilateral mastectomy under midazolam and opioid-based anaesthetic protocols. Continuous endoscopic monitoring allowed direct visualisation of reflux events throughout the remainder of the surgical procedure (from approximately 45 min after induction onward), providing detailed characterisation of reflux severity and oesophageal findings. However, because endoscopy was initiated after reflux had already occurred in all cases, this study could not characterise the timing of reflux onset.

## Materials and methods

### Ethical approval

This prospective observational study was approved by the Animal Use Ethics Committee of the Federal University of Pará (CEUA nº 2741260522). Informed consent was obtained from all dog owners.

### Study design and animals

Twelve female dogs undergoing unilateral mastectomy at the University Veterinary Hospital between May 2022 and July 2023 were enroled. Inclusion criteria were clinical indication for total unilateral mastectomy and owner consent. No formal exclusion criteria were defined a priori. This decision reflects the preliminary, real-world nature of the study but is acknowledged as a methodological limitation, as predefined exclusion criteria would have strengthened the study design by minimising selection bias and reducing the risk of including animals with subclinical pre-existing reflux. A retrospective review of medical records was subsequently performed to assess for known predisposing factors. This review revealed that none of the enrolled dogs had a documented history of hiatal hernia, chronic vomiting, regurgitation, or pre-existing gastric disease, and no dog had received medications known to affect gastrointestinal motility (e.g., metoclopramide, cisapride) or LOS tone (e.g., prokinetics, anticholinergics) in the 7 days prior to anaesthesia. However, the absence of exclusion criteria leaves some uncertainty regarding potential subclinical reflux prior to anaesthesia. The heterogeneity in body weight (4.7–31.9 kg) reflects the typical clinical caseload of a university veterinary hospital and was not an exclusion criterion, as no a priori weight restriction was imposed. Caution is therefore warranted when interpreting the results in this heterogeneous population.

The following time intervals were recorded: anaesthesia time – defined as the period from orotracheal intubation to extubation; surgical time – defined as the period from skin incision to completion of skin suturing; and time from induction to endoscopy start – defined as the period from propofol injection to insertion of the endoscope into the oesophagus.

### Anaesthetic protocol

The animals were fasted for 12-hours for food and 4 to 6-hours for water. Premedication consisted of intramuscular midazolam (0.1–0.3 mg/kg; Dormire^®^, Cristália, Brazil) with morphine (0.5-1.0 mg / kg; Dimorf^®^, Cristália, Brazil) or tramadol (2.0–4.0 mg / kg; Tramadon^®^, Cristália, Brazil). Anaesthesia was induced with intravenous propofol (4.0–6.0 mg/kg; Propovan^®^, Cristália, Brazil), followed by orotracheal intubation and maintenance with isoflurane (Isoforine^®^, Cristália, Brazil) in 100% oxygen at a fresh gas flow of 1–2 L/min. All dogs received local tumescent anaesthesia (lidocaine 7 mg/kg, adrenaline 0.12–1.2 ml/animal, saline 15 ml/kg).

Time intervals and catheterisation: Premedication was administered intramuscularly immediately after placement of a 22-gauge cephalic venous catheter under manual restraint. Venous catheterisation upon hospital admission is routine practice at our institution, as it provides immediate vascular access in case of emergency or adverse reaction to premedication, avoids the difficulty of catheterising a sedated patient, and allows the same catheter to be used for intravenous induction agents. The interval between premedication and induction of anaesthesia was 15–20 min, allowing adequate onset of sedative effects.

Intraoperative monitoring: Throughout anaesthesia, patients were monitored using a multiparameter monitor (Dixtal^®^, DX2020, Brazil) recording heart rate (ECG), respiratory rate (capnography and thoracic excursion), non-invasive oscillometric blood pressure (cuff placed on the antebrachium), peripheral oxygen saturation (SpO₂), end-tidal carbon dioxide (ETCO₂), and oesophageal temperature. Isoflurane administration was adjusted to maintain a surgical anaesthetic plane characterised by loss of palpebral reflex, absence of purposeful movement, and stable cardiovascular parameters (heart rate and mean arterial pressure within 20% of baseline). The vaporiser setting ranged from 1.2% to 2.0% end-tidal concentration. All dogs were mechanically ventilated using a rebreathing circuit (circle system) with tidal volume set at 10–15 mL/kg and respiratory rate adjusted to maintain ETCO₂ between 35 and 45 mmHg. Fraction of inspired oxygen (FiO₂) was 1.0 (100% oxygen) throughout the procedure.

Fluid therapy: Intraoperative fluid therapy consisted of lactated Ringer’s solution (Ringer com Lactato^®^, Fresenius Kabi, Brazil) administered at a rate of 5–10 mL/kg/h via a volumetric infusion pump.

Intraoperative analgesia assessment and rescue: Analgesia was assessed intraoperatively based on cardiovascular responses to surgical stimulation (heart rate and mean arterial pressure increases > 20% above baseline). No dog required rescue intraoperative analgesia beyond the established protocol, as the combination of systemic opioids, local tumescent anaesthesia (lidocaine 7 mg/kg with adrenaline), and isoflurane provided adequate surgical tolerance.

Postoperative management: After extubation, all dogs received meloxicam (0.2 mg/kg subcutaneously; Maxicam^®^, Ourofino, Brazil) for postoperative analgesia. Antibiotics (cefazolin 22 mg/kg intravenously at induction, repeated every 90 min) were administered perioperatively. In cases where GER was detected endoscopically, no specific anti-reflux therapy (e.g., sucralfate, omeprazole, maropitant) was administered during anaesthesia, as the study was observational and intervention was not part of the protocol. However, the dog with pre-existing oesophagitis received oral omeprazole (1 mg/kg once daily for 14 days) postoperatively.

The interval between induction and the start of oesophageal endoscopy corresponded to the time required for the administration of local tumescent anaesthesia and surgical field preparation with antisepsis and draping.

### Surgical procedure

The patients were placed in the supine position with unilateral radical mastectomy performed with incisions made parallel to the affected mammary chain. The subcutaneous tissue was dissected from the thoracic mammary glands to the abdominal and inguinal glands. The superficial caudal epigastric vein was ligated with 25-PGCL poliglecaprone (Bioline^®^, São Paulo, Brazil) and removed with the mammary chain and inguinal lymph node. The incision was closed in two layers with an intradermal suture using PGCL, followed by skin closure with interrupted 3‑0 nylon sutures (Procare^®^, Rio de Janeiro, Brazil).

### Oesophagoscopy

Immediately before the surgical incision, a flexible endoscope (150 cm in length, 8.9 mm diameter) was inserted orally for continuous observation of the esophagogastric junction. Air insufflation was used sparingly and only as necessary to maintain visualisation of the oesophageal lumen, with the insufflation pump set to the minimum pressure required (approximately 5–8 mmHg). The endoscope tip was positioned 2–3 cm proximal to the LOS, and insufflation was temporarily suspended whenever LOS relaxation or reflux events were being assessed. When lens fogging or soiling occurred, a minimal volume (1–2 mL) of sterile water was instilled via the working channel to clean the lens, followed by gentle suction to remove excess fluid. No water was instilled with the tip positioned at the LOS to avoid hydrostatic pressure-induced cardia opening. The endoscope was never advanced into the stomach until after spontaneous reflux events had been documented, as gastric intubation invariably requires LOS passage and induces transient sphincter relaxation. All endoscopic procedures were performed by a single experienced endoscopist (P.S.R.) to ensure consistency of technique. The lower oesophageal sphincter and Gastro‑oesophageal reflux were endoscopically assessed throughout the surgical procedure. Reflux was classified as: GERD0 (no), GERD1 (mild, reaching the distal oesophagus) or GERD2 (marked, reaching the middle/cranial oesophagus).

Endoscopic equipment: A flexible video endoscope (Model ENDOVISION, GDI^®^, São Paulo, Brazil) was used, measuring 150 cm in length with an outer diameter of 8.9 mm and a 2.8 mm working channel.

Endoscope positioning and stabilisation: Immediately before the surgical incision, the endoscope was introduced orally and advanced under direct visualisation to the level of the lower oesophageal sphincter (LOS), located approximately 40–45 cm from the dental arcade depending on patient size. The endoscope tip was positioned 2–3 cm proximal to the LOS to avoid mechanical cardia opening while allowing continuous observation of the oesophagogastric junction. The endoscope was stabilised by securing the insertion tube to the operating table using a non-penetrating clamp and foam padding to prevent dislodgement without applying traction.

Image acquisition and assessment: Continuous video recordings were captured throughout the procedure using a digital video processor (EPK-1000, Pentax Medical^®^, Tokyo, Japan) and stored for subsequent analysis. Endoscopic images were independently reviewed by two experienced endoscopists (P.S.R. and H.J.G.) who were blinded to the opioid protocol used. Disagreements were resolved by consensus with a third reviewer (P.P.M.T.).

Evaluation of the lower oesophageal sphincter: The LOS was assessed continuously for evidence of spontaneous relaxation, defined as visible opening of the sphincter with or without passage of gastric contents into the oesophagus. For the purposes of this study, LOS relaxation was recorded as either present (visible opening) or absent (sustained closure). The previously described three-point grading system (none, partial, complete) was considered but not applied, as consistent distinction between partial and complete relaxation proved unreliable during live endoscopy. Cardia opening induced by endoscopic pressure was avoided by maintaining the tip position proximal to the LOS and advancing only after documentation of spontaneous reflux events.

### Statistical analysis

Continuous variables (age, weight, duration of surgery, anaesthesia time, endoscopy start time) were described using descriptive statistics (mean, standard deviation, median, and range). Reflux grade frequencies were reported as proportions. Data are therefore presented descriptively only. All analyses were performed using BioEstat version 5.3 (Instituto Mamirauá, Brazil).

## Results

The studied population comprised twelve female dogs from following breeds: mixed-breed (*n* = 7), German shepherd (*n* = 1), Poodle (*n* = 1), Rottweiler (*n* = 1), Siberian Husky (*n* = 1) and French Bulldog (*n* = 1), of which eight received midazolam combined with morphine and four received midazolam combined with tramadol for preanaesthesia medication. Among the eight dogs receiving morphine, the mean dose was 0.78 ± 0.21 mg/kg (range 0.5–1.0 mg/kg). Among the four dogs receiving tramadol, the mean dose was 3.1 ± 0.8 mg/kg (range 2.0–4.0 mg/kg). Midazolam was administered at a mean dose of 0.22 ± 0.06 mg/kg (range 0.1–0.3 mg/kg) across all animals. The mean duration of the surgery was 104 ± 32 min (range 47–150 min), the mean anaesthesia time was 158 ± 33 min (range 90–212 min), and the mean time from induction to the start of the oesophageal procedure was 45 ± 19 min (range 21–90 min).

**Fig. 1:**
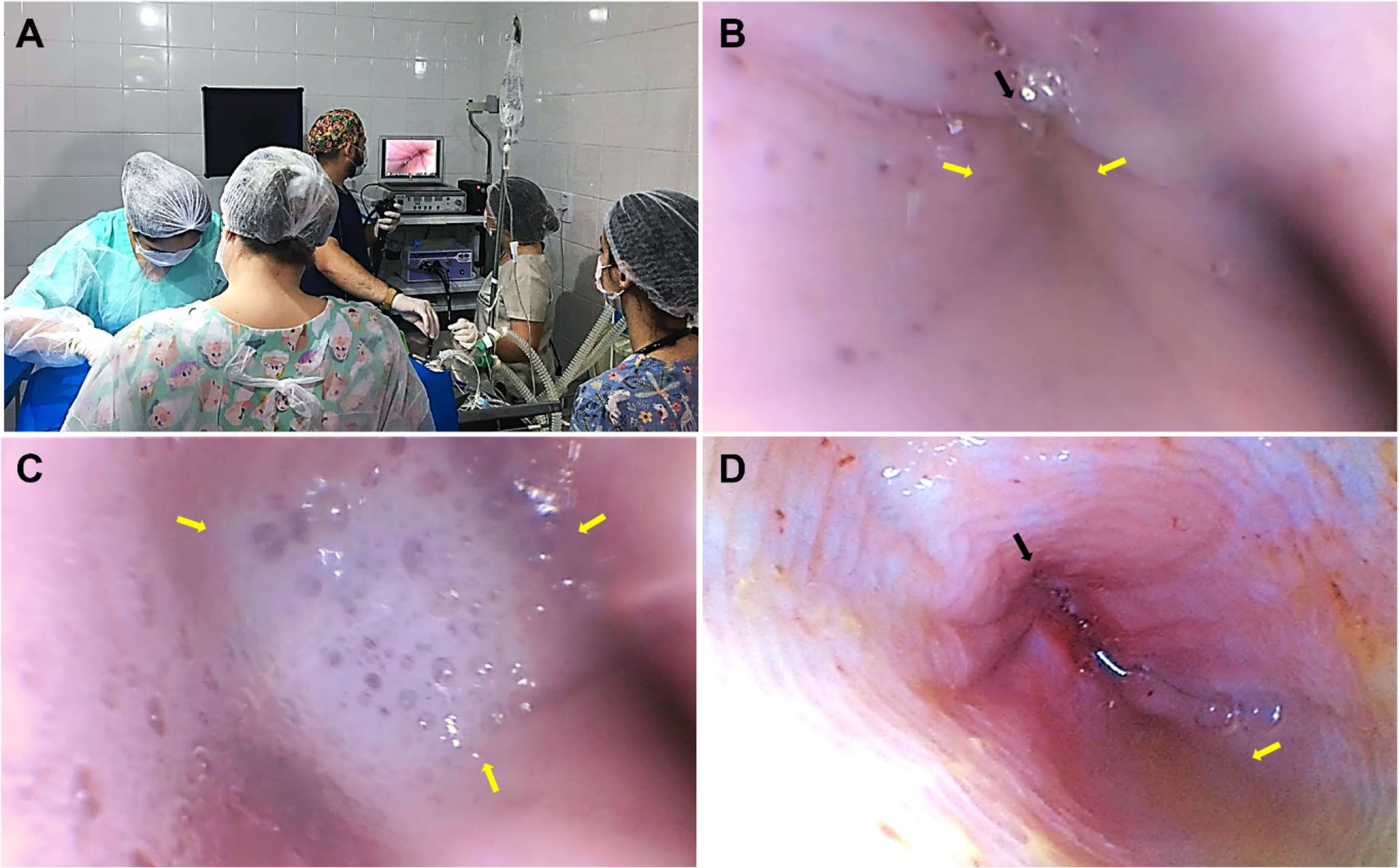
Intraoperative endoscopic evaluation of Gastro‑oesophageal reflux in dogs undergoing mastectomy. (**A**) Perform an endoscopic examination during unilateral mastectomy. (**B**) GER1 classification showing mild reflux (yellow arrows) reaching the distal oesophagus with slight relaxation of the lower esophageal sphincter (black arrow). (**C**) GER2 classification that shows marked reflux with accumulation of secretion (yellow arrows) extending to the cranial third of the oesophagus. (**D**) GER2 classification in a patient with pre-existing endoscopic oesophagitis, revealing enanthema and inflammation in the distal oesophagus and lower oesophageal sphincter (black arrow)

Gastro‑oesophageal reflux was identified in the 12 female dogs (100%) through esophagoscopy (Fig. 1A). LOS relaxation (defined as visible opening of the sphincter) was observed in all 12 cases (100%). Due to the challenges of consistently distinguishing partial from complete relaxation during live endoscopy, further gradation of LOS relaxation severity was not performed. Of these, seven (58%) were classified as GER1 (mild reflux reaching the distal oesophagus) (Fig. 1B) and five (42%) as GER2 (marked reflux reaching the middle or cranial oesophagus) (Fig. 1C). The refluxate was visually characterised as follows: volume was subjectively graded as small (< 1 mL visible pooling), moderate (1–5 mL), or large (> 5 mL). Consistency was classified as liquid (*n* = 9, 75%), mucoid (*n* = 2, 17%), or particulate/mixed with food residue (*n* = 1, 8%). Colouration was predominantly clear to yellow-green (bilious appearance) in 10 dogs (83%) and blood‑tinged/hematic in 2 dogs (17%). These characteristics remained stable throughout the intraoperative period in all cases, with no progression in volume or change in composition observed during the endoscopic inspection.

One dog (mixed-breed, 11 years, 21 kg) presented endoscopic evidence of pre-existing oesophagitis, characterised by enanthema and inflammation in the distal oesophagus and lower oesophageal sphincter (Fig. 1D). This dog was not excluded a priori because the study initially applied no exclusion criteria.

No episodes of vomiting occurred after premedication. An episode of regurgitation was observed during the intraoperative period.

Comparison of reflux grades between opioid groups showed that among dogs receiving morphine, 4/8 (50%) presented GER1 and 4/8 (50%) presented GER2. Among dogs receiving tramadol, 3/4 (75%) presented GER1 and 1/4 (25%) presented GER2. Within the morphine group (*n* = 8), dose distribution was as follows: 0.5 mg/kg (*n* = 2), 0.7 mg/kg (*n* = 1), 0.8 mg/kg (*n* = 2), 0.9 mg/kg (*n* = 1), and 1.0 mg/kg (*n* = 2). Within the tramadol group (*n* = 4), doses were: 2.0 mg/kg (*n* = 1), 3.0 mg/kg (*n* = 1), 3.5 mg/kg (*n* = 1), and 4.0 mg/kg (*n* = 1). The wide dose range reflects clinician discretion based on patient body condition and perceived pain risk. No formal statistical comparison of mean doses between groups was performed, as the small sample size and unequal group distribution would render any such analysis underpowered. The small sample size also precludes any definitive conclusions about dose-response relationships with GER severity.

## Discussion

Our 100% GER incidence exceeds previous reports of 44.1% (Flouraki et al. 2022a), 45% (Wilson et al. 2005), and 38% (Galatos and Raptopoulos 1995). Higher detection in our study may reflect continuous endoscopic monitoring (Weigt et al. 2007), synergistic midazolam-opioid LOS relaxation, and supine positioning abolishing gravitational barrier (Wilson et al. 2005). However, since reflux was visualised prior to surgical incision, findings reflect the anaesthetic period, attributable to opioids, midazolam, isoflurane, and positioning, not surgery itself (Costa et al. 2021). This interpretation remains speculative, but absence of a control group precludes definitive attribution.

The absence non-surgical control group (cannot attribute GER to any specific factor), small sample size (*n* = 12, limits power and generalisability), and potential endoscopic artefact. Endoscopy itself may influence LOS tone: air insufflation triggers transient relaxations, and water instillation may open the cardia (Mittal et al. 1995). We minimised artefacts via minimal insufflation, suspending insufflation during assessment, and avoiding water at the LOS. Reflux occurred without active insufflation, suggesting findings are not artefactual, though endoscope presence is a shared limitation (Weigt et al. 2007). The dilated oesophagus in Fig. 1 reflects minimal insufflation, not pathology. Insufflation-induced LOS reduction, 5–10%, is minor compared to opioid-induced reduction, 40–60% (Kraichely et al. 2010).

Opioid receptors (µ, δ, ĸ) in gastrointestinal smooth muscle (Ghosh et al. 2022) mediate motor effects. Their presence in the human LOS may explain relaxation. Opioids also cause non-propulsive contractions, increasing reflux likelihood (Costa et al. 2021). GER cannot be attributed exclusively to opioids, as isoflurane and patient positioning also contribute. Our findings are consistent with opioid‑induced LOS relaxation, but causality cannot be established. All patients refluxed regardless of opioid type, consistent with Flouraki et al. (2022a) showing no difference between opioids. pH monitoring can show has false negatives and misses non-acid reflux (Weigt et al. 2007; Johnson 2014). Endoscopy allowed direct visualisation and avoided cardia opening by deferring stomach access (Weigt et al. 2007). We did not perform pH monitoring due to equipment unavailability. Future studies should use multimodal monitoring.

Endoscopy began 45 min post-induction (pre-incision) due to tumescent anaesthesia (15–20 min) and field preparation (20–25 min). We cannot determine if GER occurred immediately after induction, a period relevant to aspiration pneumonia risk (Costa et al. 2021; Flouraki et al. 2022b). One dog had pre-existing oesophagitis, a potential confounder, though universal reflux persisted without this case. Reflux commonly causes oesophagitis, reduced sphincter tone may aggravate prior episodes (Johnson 2014; Viskjer and Sjöström 2017; Costa et al. 2021). Maropitant does not prevent GER (Johnson 2014), high-dose metoclopramide shows no significant reduction (Wilson et al. 2006), and cisapride (with esomeprazole) is unavailable in Brazil (Zacuto et al. 2012). No protocol fully prevents intraoperative GER and the clinical vigilance remains essential.

The absence of a non‑surgical control group, which precludes attributing gastroesophageal reflux specifically to mastectomy versus anaesthesia itself; the small sample size (*n* = 12), limiting generalisability and precluding definitive subgroup or dose‑response analyses; and the lack of predefined exclusion criteria, introducing potential selection bias and uncertainty regarding subclinical pre‑existing reflux, even though retrospective review identified no known predisposing factors. We also recognise that endoscopic artefact (air insufflation and endoscope presence) may influence lower oesophageal sphincter tone, despite our measures to minimise this (minimal insufflation, suspension during assessment, no water instillation at the sphincter). Additional limitations include the delayed start of endoscopy (reflux already present by 45 min, so we cannot comment on events immediately after induction, a period relevant to aspiration risk); the unavailability of pH monitoring, limiting detection to visible reflux only; a heterogeneous population; no measurement of intra‑abdominal pressure to assess the influence of positioning or surgical retraction; and no objective anaesthetic depth monitoring. All limitations are presented without underplaying their impact: we clearly state that the study is preliminary and hypothesis‑generating, and that findings should be interpreted with caution.

This study cannot establish causality due to non-standardised dosing, lack of randomisation, and no non-opioid control group. Association is consistent with, but does not prove, causality. Because reflux preceded incision, findings reflect the anaesthetic period (opioids, midazolam, isoflurane, supine positioning), not surgery itself. Additional limitations such as the heterogeneous population, no predefined exclusion criteria (potential selection bias and uncertainty regarding subclinical reflux), no intra-abdominal pressure measurement, and no objective anaesthetic depth monitoring. Findings are preliminary and hypothesis-generating. Intraoperative vigilance remains essential. These results reinforce intraoperative vigilance during prolonged anaesthesia and advocate for randomised trials comparing opioid-based to non-opioid multimodal protocols with non-surgical controls.

## Data Availability

The data that support the findings of this study are available from the corresponding author upon reasonable request.
