# Correction: Intraoperative gastroesophageal reflux in dogs undergoing mastectomy under opioid-based protocols: a preliminary endoscopic study

**DOI:** 10.1007/s11259-026-11342-w

**Published:** 2026-06-26

**Authors:** Priscila dos Santos Ribas, Heytor Jales Gurgel, Gabriela Melo Alves dos Santos, Verena Siqueira da Silva, Francisco Décio de Oliveira Monteiro, Sheyla Farhayldes Souza Domingues, Roberta Martins Crivelaro Thiesen, Roberto Thiesen, Pedro Paulo Maia Teixeira

**Affiliations:** 1 Veterinary Medicine Institute, Federal University of Pará, Castanhal, Pará Brazil; 2 Federal Institute of Tocantins (IFTO), Campus Araguatins, Araguatins, Tocantins Brazil

**Correction: Veterinary Research Communications (2026) 50:348**

**https://doi.org/10.1007/s11259-026-11290-5**

In the original version of this article, the given and family names of Priscila dos Santos Ribas were incorrectly structured. The name was displayed correctly in all versions at the time of publication.

The original article has been corrected.

